# Prevalence of Overweight and Influence of Out-of-School Seasonal Periods on Body Mass Index Among American Indian Schoolchildren

**Published:** 2008-12-15

**Authors:** Derek T Smith, R. Todd Bartee, Christopher M. Dorozynski, Lucas J. Carr

**Affiliations:** Division of Kinesiology and Health, College of Health Sciences, University of Wyoming. Dr Smith is also affiliated with the Department of Zoology and Physiology, College of Arts and Sciences, University of Wyoming; University of Wyoming, Laramie, Wyoming; University of Wyoming, Laramie, Wyoming; University of Wyoming, Laramie, Wyoming

## Abstract

**Introduction:**

The prevalence of overweight and obesity among American Indian youth may be 2 to 3 times higher than the national average. Whether weight gain during discrete out-of-school periods is occurring and contributing to the prevalence of overweight and obesity in this population is unknown.

**Methods:**

We obtained repeated cross-sectional body mass index (BMI) samples from third-, fourth-, fifth-, seventh-, and eighth-grade boys and girls who reside on the Wind River Indian Reservation in central Wyoming. We collected measures at the beginning of 2 school years (N = 251), during 2 holiday breaks (N = 226), and during 1 summer recess (N = 141). We determined prevalence of normal weight and overweight among participants by grade level, and we calculated paired comparisons of BMI, BMI *z* score, and weight status during the holiday breaks and summer recess.

**Results:**

Combined prevalence of at risk for overweight and overweight was 62.0% for boys and 56.6% for girls. For fifth-grade girls, significant increases in BMI (*P* = .01) and *z* score (*P* < .001) occurred over the holiday break. BMI increased significantly over the summer among third- and fifth-grade girls and among fourth-grade boys, but changes in *z* scores were nonsignificant. We observed an increase in weight status by out-of-school time in BMI (*P* < .001) for schoolchildren at or above the 85th BMI percentile over the summer recess, but corresponding *z* scores did not change.

**Conclusion:**

Prevalence of overweight among American Indian schoolchildren was higher than national estimates and higher than the prevalence in other similarly aged American Indian youth. Increases in BMI during out-of-school periods are likely due to normal growth, except among fifth-grade girls.

## Introduction

During the past 20 years, the combination of decreased physical activity levels and an increase in unhealthy eating habits has contributed to the doubling of the percentage of overweight children and adolescents (1). Evidence indicates disparities in overweight among racial/ethnic groups (2); the prevalence of overweight and obesity among American Indian youth may be 2 to 3 times greater than the national average (3-5). Inactivity-related health problems, such as childhood obesity, are recognized as national health priorities, and it has been argued that the US health care system is unprepared to deal with these challenges, particularly with regard to understudied racial/ethnic minorities (6-8).

Childhood overweight and obesity (9) and cardiovascular disease risk factors (10,11) may persist into early adulthood. The multifactorial problem of childhood overweight and obesity is in part influenced by genetics, rearing and school environment, availability and quality of foods, and energy expenditure (physical activity) (12-15). To quantify the extent of childhood overweight during the temporal fluctuations in growth and development (16,17) and to provide a measure of the efficacy of obesity prevention and intervention, monitoring of age- and sex-normalized body mass index (BMI) has been promoted by the American Academy of Pediatrics (18). Monitoring of BMI over discrete periods of potential energy imbalance (excess intake combined with deficient expenditure) during childhood and adolescence has not been extensively conducted. Transient and discrete periods of developmentally inappropriate weight gain may contribute to the increasing prevalence of childhood overweight and obesity (1).

In adults, the holiday season from late November through early January may be a period of potential weight gain in which energy imbalance may contribute to long-term weight retention (19,20). In a convenience sample of adults, an average holiday weight gain of 0.37 kg was reported with a net weight gain from September or October through February or March of 0.48 kg, equivalent to approximately 1 pound (20). Results from another study showed a weight gain of 0.4 kg and 0.6 kg in male and female college students, respectively, over the Thanksgiving break (21). Both studies similarly concluded that the retention of the short-term weight gain may have long-term health implications (20,21). In children, the holiday season is a period during which weight gain may occur. Another period that may be conducive to school-based obesity prevention programs in minority populations (22-24) is the summer recess. Over time, periods of being out of school, during which weight may be gained and retained, may cumulatively contribute to the prevalence of childhood overweight and obesity and the ensuing health complications associated with these conditions.

To our knowledge, no studies have investigated discrete periods of weight gain or increase in BMI among minority populations, specifically American Indian schoolchildren. Moreover, the prevalence of overweight in American Indian schoolchildren residing on the Wind River Indian Reservation has only been informally reported through schools and local and state health authorities. Several factors highlight the need for more detailed surveillance and follow-up in these youth: 1) underreporting of overweight and obesity prevalence in this population, 2) absence of attention to the out-of-school periods in this geographic region, 3) potential influence of inclement weather, and 4) geographic isolation (rural location) contributing to reduced opportunities for physical activity. The combination of the latter 2 factors may lead to increased indoor confinement without the opportunity to engage in physical activity and contribute to a higher prevalence of overweight and obesity in American Indian youth compared with national estimates (5,25).

As part of an ongoing community-based participatory research (CBPR) project between investigators at the University of Wyoming and a school community on the Wind River Indian Reservation, the school community developed a goal of building a culture of physical activity to prevent childhood overweight and its complications. Since 2005, BMI of schoolchildren has been monitored in select grade levels of 1 American Indian school district as part of the CBPR projects' physical activity intervention. Determining the prevalence of overweight among the schoolchildren and identifying periods of developmentally inappropriate weight gain was a necessary first needs assessment. Therefore, the purposes of this study were to 1) determine and report the prevalence of children at a normal weight, children at risk for overweight, and children who were overweight, in a convenience sample of American Indian schoolchildren; and 2) determine whether increases in BMI and *z* score occurred during seasonal out-of-school periods (holiday breaks and summer recess).

## Methods

### Participants and study design

Participants were Northern Arapaho and Eastern Shoshone schoolchildren (third, fourth, fifth, seventh, and eighth grades) attending an elementary and middle school on the Wind River Indian Reservation in central Wyoming. The cooperating school district excluded sixth graders because of the absence of a school team leader (adult) to represent this grade and because the sixth grade is the transition between elementary and middle school. First, repeated cross-sectional data on BMI, age (nearest month), and sex were collected to determine the prevalence of overweight at the beginning of 2 consecutive school years (September 5th and 6th, 2005, and September 4th and 5th, 2006) in 251 schoolchildren. Second, to determine whether a significant change in BMI or weight gain occurred over the 2 holiday breaks (December through January of 2005-2006 and 2006-2007), measures were repeated immediately before and after these periods in 2 subsets of schoolchildren (N = 226): 1) December 14th through 22nd, 2005, and January 4th through 12th, 2006 (N = 80); and 2) December 13th through 21st, 2006, and January 8th through 16th, 2007 (N = 146). All holiday break comparisons were made within school grade level and are for unique children (ie, fifth-grade children measured over the 2005 holiday break were different children than the fifth-grade children measured over the 2006 holiday break), with the exception of less than 3% of situations in which a child was retained. Third, BMI measures were repeated immediately before and after the summer 2007 recess (May 23rd-24th and August 29th-30th) in all available schoolchildren (N = 141). (Although most participating schoolchildren matriculated over the summer of 2007, all summer recess data refer to children’s May 2007 school grade level.) Research protocols were approved by the institutional review board at the University of Wyoming, Laramie, Wyoming, and consent for participation was obtained from the school district according to its established procedures.

### Measurements

We measured weight to the nearest 0.1 pound using a calibrated digital scale (Seca Model 780, seca gmbh & co, Hamburg, Germany) and measured height to the nearest 0.25 inch by using a height rod (Seca Model 220, seca gmbh & co, Hamburg, Germany), without shoes and excess clothing. The same trained investigators performed the repeated height and weight measures. We converted height and weight to SI units, and we calculated BMI as follows: BMI (kg/m^2^) = weight (kg)/height (m^2^). BMI was normalized for each child's age (nearest month) and sex to determine BMI percentile, according to the Centers for Disease Control and Prevention's BMI-for-age growth charts (26), and BMI *z* score. Normalized BMI percentile was used to classify each child's weight status, where *normal* is below the 85th percentile (this category may include children who are underweight), *at risk for overweight* is 85th to less than the 95th percentile, and *overweight* is greater than or equal to the 95th percentile (26). Age- and sex-normalized BMI and *z* score were the primary outcome variables of interest for the out-of-school comparisons.

### Statistical analyses

We combined data from the 2005 and 2006 academic years and data from the respective holiday seasons to increase the power of our analyses. Combining data allowed new participants — third- and sixth-grade students in 2005 who became part of the sample in 2006 as fourth- and seventh-grade students, respectively — to be incorporated. Before data were combined, we conducted preliminary analyses (*t *test and χ^2^; data not reported) to determine whether the prevalence of overweight and obesity was similar by grade level for the 2 years (2005 and 2006). We repeated this preliminary procedure for the 2 corresponding holiday seasons, and no differences between academic years or holiday seasons were identified. Prevalence data reported for the combined years included BMI, age-normalized BMI percentile, *z* score, and weight status (normal weight in 1 category and at risk for overweight combined with overweight in another category) for all schoolchildren. We report prevalence data for school grade level (third, fourth, fifth, seventh, and eighth) with corresponding ages. Within grade level, sex comparisons for age, BMI, BMI percentile, *z* score, and select grade-to-grade comparisons were analyzed by using *t *tests. BMI, height, weight, and *z* scores before and after the holiday break and the summer recess were analyzed by 1-tailed paired *t *test to test the hypothesis that increases occur over the holiday break and summer recess. Sex-combined and sex-dependent comparisons of before and after out-of-school periods were made within each grade level and for combined grade levels (summer only). The holiday break and summer recess were treated separately. To determine whether an interaction existed between weight status and change in BMI and change in *z* score for the out-of-school periods, the at risk for overweight and overweight categories were combined, yielding a weight status category of BMI ≥85th percentile. Two-way repeated measures of analysis of variance (weight status by time) were performed with post hoc testing (Tukey) when indicated by a significant F score. Statistical significance was set at *P* < .05, and all analyses were performed using SigmaStat version 3.11 (Systat Software, Inc, San Jose, California) and SPSS version 16.0 (SPSS Inc, Chicago, Illinois).

## Results

### Weight status findings

Overall, no significant within-grade sex differences were found, except for eighth-grade boys who were significantly older than their female peers (Table 1). Seventh-grade boys were the only group with a mean age- and sex-normalized BMI percentile greater than the at risk for overweight cut point (≥85th percentile; Table 1). Most schoolchildren (86.8%) were either normal weight or overweight (Table 2). When prevalence of at risk for overweight and overweight were combined, 62.0% of boys and 56.6% of girls were at or above the 85th percentile.

### Body mass index over the holiday break

Over the holiday break, BMI did not change significantly in the third, fourth, seventh, and eighth grades, and the corresponding BMI *z* scores were similarly nonsignificant. From December to January in the fifth grade sex-combined sample (N = 67), a small but significant increase in BMI (*P* = .02) was found (data not shown). Sex-dependent analyses revealed that the increase in BMI was isolated to girls (*P* = .01), and the *z* score increased significantly for these fifth-grade girls (*z* score_before_ = 1.28, SD = 0.91 vs *z* score_after_ = 1.33, SD = 0.88; *P* < .001). In fifth-grade students, weight increased significantly among boys (*P* = .01) and girls (*P* < .001) and was not different between sexes (*P* = .47). Similarly, height increased in fifth-grade boys and girls, but the growth was only significant among boys (*P* < .001), likely contributing to the isolated increase in BMI and *z* score among fifth-grade girls. For independent or combined grade levels, no associations were found between weight status (BMI ≥85th percentile) and change in BMI or *z* score over the holiday break for sex-combined samples.

### Body mass index over the summer recess

**Figure 1:**
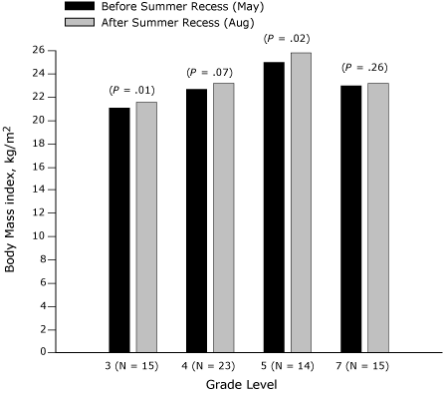
Body mass index (BMI, kg/m^2^) before and after summer recess in third-, fourth-, fifth-, and seventh-grade girls, Wind River Indian Reservation, Wyoming, 2007.

**Figure 2:**
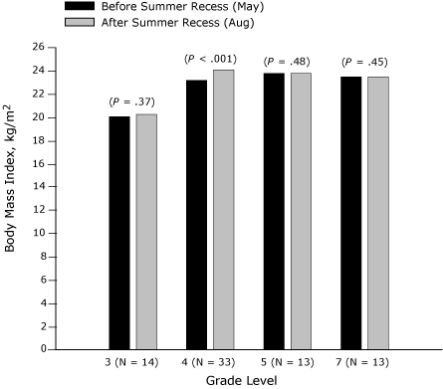
Body mass index (BMI, kg/m^2^) before and after summer recess in third-, fourth-, fifth-, and seventh-grade boys, Wind River Indian Reservation, Wyoming, 2007.

The effect of summer recess (May to August 2007) on BMI of third-, fourth-, fifth-, and seventh-grade students is presented in Figure 1 (girls) and Figure 2 (boys). Among seventh-grade boys and girls, no significant change in BMI was found, but in each of the other grades a significant increase for 1 sex was found. BMI increased significantly among third-grade girls (*P* = .01) and fifth-grade girls (*P* = .02) (Figure 1). Among third-grade girls, a significant weight increase (*P* < .001) was found over the summer recess, although height increased nonsignificantly. Similarly, among fifth-grade girls, a significant weight increase was found (*P* = .04), although height increased nonsignificantly. BMI and weight increased significantly only among fourth-grade boys (*P* < .001), although height increased insignificantly.

**Table:** 

**Table:** 

**Figure 3:**
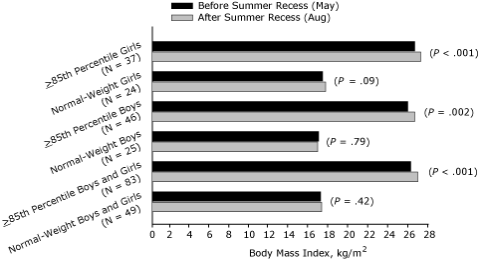
Age- and sex-adjusted body mass index (BMI, kg/m^2^) before and after summer recess in third-, fourth-, fifth-, and seventh-grade students, Wind River Indian Reservation, Wyoming, 2007.

**Figure 4:**
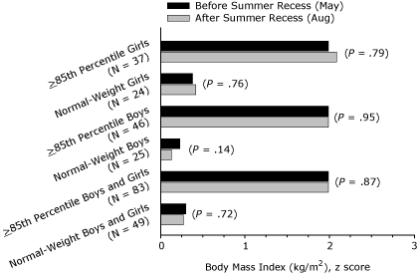
Body mass index (BMI, kg/m^2^)* z* score before and after summer recess in third-, fourth-, fifth-, and seventh-grade students, Wind River Indian Reservation, Wyoming, 2007.

A significant association (*P* = .02) was found between weight status (normal weight vs BMI ≥85th percentile) and change in BMI over the summer recess for the sex-combined sample (Figure 3). BMI increased significantly in the sex-combined sample for students at or above the 85th percentile (*P* < .001) over the summer recess, whereas no change was observed for their normal-weight peers (Figure 3). Figure 3 shows that, for boys and girls independently at or above the 85th percentile, a significant increase in BMI was found over the summer recess, but no change in BMI for normal-weight boys or normal-weight girls was found. The observed interactions and changes in summer recess BMI for the boys, girls, and the sex-combined sample were not accompanied by significant increases in BMI *z* score or *z* score interactions (weight status by time) for any group (Figure 4).

**Table:** 

**Table:** 

## Discussion

### Weight status

To our knowledge, this is the first formal study of the prevalence of overweight and at risk of overweight in American Indian children aged 7 to 14 years residing on the Wind River Indian Reservation in central Wyoming. The prevalence of overweight reported here differs from a recent study on American Indian schoolchildren residing on or near the Aberdeen Area of the Indian Health Service (North Dakota, South Dakota, Nebraska, and Iowa) (4). Zephier et al defined obesity as a BMI greater than the 95th percentile, consistent with our overweight classification. However, they estimated that 31.5% of boys and 27.2% of girls were obese (4), lower percentages than the 50.4% of boys and 41.8% of girls who were overweight in our sample. The prevalence of overweight that we observed should be viewed in context of the 38.0% of boys and 43.4% of girls who are at a normal weight.

Most children in our sample (86.8%) were either at a normal weight or were overweight. The observed distribution of schoolchildren in the normal weight or overweight categories is similar to that reported for rural public school students in Oklahoma, 40.3% of whom were American Indian. Among second-grade through eighth-grade students, the prevalence of normal weight was approximately 51%, and the prevalence of overweight was 31%, totaling 82% of the sample (27). Whether these patterns of weight status distribution are unique to specific rural environments, race/ethnicity, or both is unclear, but they are contrary to the 2003-2004 national youth estimates, showing the highest prevalence in the at risk for overweight category in children aged 6 to 11 years (37.2%) and aged 12 to 19 years (34.3%) (2). Furthermore, approximately 60% of the schoolchildren in our study were at or above the 85th BMI-for-age percentile, which underscores the seriousness of the epidemic and confirms evidence from others indicating that the rates of overweight among American Indian youth are greater than those of the overall US youth population (5,25). The high prevalence of overweight reported for these American Indian schoolchildren has clinical implications because childhood overweight increases the potential for acquiring additional cardiometabolic disease risk factors and disease at an early age (28) and because multi-racial cohort studies demonstrate a strong association between childhood overweight and early maturation and menarche (29-31).

### Influence of out-of-school time on BMI and weight status

Increases in BMI by sex and grade level (age) were observed during the holiday break and summer recess. One limitation of our study is that it is only a single snapshot of the influence of out-of-school time on developmentally unexpected increases concomitantly of BMI and weight. However, the significant increases found among fifth-grade girls for BMI (8.6%) and *z* score (3.6%) over the holiday season should not be disregarded because they equate to a weight gain of approximately 1.5 pounds in fewer than 30 days. This is consistent, albeit in a much older female population, with the finding that college-age women experienced an approximate 0.9-pound increase in weight during the Thanksgiving holiday and suggested that the potential long-term health consequences of retaining this weight could be significant (21). During the summer recess (approximately 3 months), we observed an increase in BMI in 3 of the 4 grade levels for either boys or girls. Similar to the holiday break findings, BMI increased again during the summer recess among fifth-grade girls; this finding indicates that, among these approximately 10-year-old girls, at least 2 out-of-school periods may be contributing to the risk for overweight or to weight gain that is outpacing the girls' linear growth. BMI also increased during the summer recess among third-grade girls and fourth-grade boys, and a trend (*P* = .07) was observed among fourth-grade girls.

The increases in BMI that we observed are both contrary and similar to evidence about patterns of physical activity during different seasons among populations of similar ages. Objectively measured physical activity levels in 5,595 English children aged 11 years revealed that they were most active during the summer season and least active during the winter (32). This evidence supports the increase in BMI over the holiday break (ie, during winter) but is contrary to the increase in BMI during summer recess. Obvious limitations to making this comparison are differences in geography and culture. Assessing whether observed out-of-school increases in BMI (ie, weight gain disproportional to vertical growth) were retained over time is not possible in a study of such short duration. Future studies should longitudinally investigate the potential for cumulative out-of-school weight gain and retention in American Indian schoolchildren because our findings suggest that this may be occurring in at least some schoolchildren, independent of sex and age.

For short-term surveillance, especially surveillance related to prevention and intervention programs, the International Obesity Task Force and others have indicated that measurement of absolute BMI provides a reasonable tracking measure of fatness (33,34). In consideration of the limitations of BMI, not restricted to growth and development, other measures, such as BMI percentile and *z* score, have been identified as reasonable accompanying or substitute measures (33,34). Absolute BMI (kg/m^2^) is appropriate and is an acceptable alternative to *z* score for assessing adiposity change over time when following children at risk for becoming obese (33). This conclusion is based in part on the demonstrated within-child *z* score variability over time, depending on the child's level of adiposity (33). Our intent was to assess both BMI and *z* score in parallel during out-of-school periods, postulating that any significant changes in both might provide stronger rationale for age- or sex-targeted intervention through the ongoing CBPR intervention in this American Indian school district.

Observed out-of-school increases in absolute BMI units may be confounded by 2 other factors. First, the age- and sex-specific increases during the holiday and summer recesses may have been influenced by seasonal fluctuations in height and weight. Rates of change in height and weight in prepubertal children are biphasic; seasonal reductions in linear growth occur from autumn to midwinter and growth spurts and increases in weight occur during the spring (16,17). The seasonal latency of height and progression of weight may have influenced the findings among fifth-grade girls. Second, a recent physical activity review of American Indian adults revealed small effect sizes for physical activity level with regard to physical environment (inclusive of weather) (35), but qualitative research has shown that weather poses a constraint to physical activity levels (summer is conducive to participating in physical activity) (36,37). Whether seasonality contributed to changes in physical activity levels of the American Indian schoolchildren, which may have reciprocally influenced weight status and weight gain during the out-of-school periods, is unknown, but this school community is tracking children's physical activity levels to investigate such implications.

The absence of parallel changes in BMI and *z* score for the out-of-school periods, in all but fifth-grade girls, does not eliminate our potential contribution to planned or previous school-based cardiovascular risk factor reduction and obesity prevention programs (23) involving underserved or minority populations, such as Pathways (22) and the Coordinated Approach to Child Health (CATCH) (24). Theoretically, our data suggest that improvements in and maintenance of BMI or weight status elicited through an in-school intervention may not be lost during the summer and holiday months when schoolchildren are probably not receiving direct or continuous intervention contact. However, this suggested absence of out-of-school relapse was recently countered in a 9-month trial of rural schoolchildren who were randomized to an in-school lifestyle physical education intervention. Improvements in body fat percentage from start to completion of the intervention showed that an almost complete relapse occurred (3.7% relative change) when children were assessed after the summer vacation (38). Moreover, fitness level (measured as VO_2_ max) diminished after the summer vacation (38). Although our findings and measures (BMI and *z* score) differed, both studies appear to be inconclusive but highlight the need to measure physical activity and fitness levels during out-of-school periods.

On the Wind River Indian Reservation, environment, inclement seasonal weather, and geographic isolation have been identified as factors that inhibit physical activity among youth, and youth on this reservation may have limited access venues for physical activity that are more common in metropolitan areas (37). A recent report from the South Carolina Rural Health Research Center found that children in rural areas (16.5%) were more likely to be obese than were children in urban areas (14.4%); additionally, more than 40% of children in rural areas did not participate in any after-school sports in 2003, and nearly half spent at least 2 hours per day using electronic entertainment media that was noneducational (39). If these statistics are representative of the rural central Wyoming region, that environment may be suspect. However, in our sample, the out-of-school discrete periods may not be a significant contributor to the prevalence of overweight in American Indian schoolchildren, with the exception of fifth-grade girls.

### Conclusion

This study is the first part an ongoing CBPR project in an American Indian school community on the Wind River Indian Reservation. We have presented evidence to more accurately describe weight status among these schoolchildren and the influence of out-of-school periods on their weight status. This information has been and is being communicated to the school district administration and other decision makers within the school community. Combined with the school's implementation of a Web-based physical activity tracking tool, these findings will help the CBPR project achieve the school community's long-term goal of creating a culture of physical activity to prevent childhood overweight and its complications. Two examples exemplify evidence of progress toward this goal. A required 15-minute physical activity (predominantly walking) session was implemented in the elementary school, and the middle school implemented and tested a year-long incentive-based physical activity program through their health course. The decision makers and team leaders of this American Indian school district are invested in and leading the way toward achieving their goal and are to be commended.

## Figures and Tables

**Table 1:** Characteristics of American Indian School Children (N = 251), Wind River Indian Reservation, Wyoming, 2005-2006

**Grade Level (n)**	Age, y, Mean (SD)	BMI, kg/m^2^, Mean (SD)	BMI Percentile^a^, Mean (SD)	BMI *z* score, Mean (SD)
**Boys**				
Third (36)	7.9 (0.4)	20.0 (4.6)	77.5 (24.9)	1.2 (1.1)
Fourth (30)	8.9 (0.4)	21.5 (4.8)	81.6 (24.5)	1.4 (1.0)
Fifth (32)	9.8 (0.5)	23.7 (6.6)	82.0 (27.3)	1.4 (1.3)
Seventh (17)	12.0 (0.4)	25.5 (6.2)	87.1 (17.4)	1.5 (0.9)
Eighth (14)	13.3 (0.7)^b^	23.6 (5.1)	76.1 (25.4)	1.1 (1.0)
**Girls**				
Third (35)	7.9 (0.4)	21.0 (4.9)	80.7 (24.9)	1.3 (1.2)
Fourth (30)	8.9 (0.4)	21.8 (5.9)	78.4 (24.5)	1.2 (1.0)
Fifth (35)	9.9 (0.5)	22.0 (4.9)	81.3 (19.8)	1.2 (0.9)
Seventh (12)	11.9 (0.5)	24.6 (7.6)	81.3 (19.5)	1.2 (0.9)
Eighth (10)	12.7 (0.5)	23.5 (4.3)	79.0 (28.2)	1.0 (0.9)

Abbreviations: SD, standard deviation; BMI, body mass index.

a Age- and sex-adjusted BMI percentile (26).

b *P* = .02 compared with girls within same grade.

**Table 2:** Prevalence of Weight Status by Sex and School Grade Level, Wind River Indian Reservation, Wyoming, 2005-2006

**Grade Level**	No. at a Normal Weight (%)^a^	No. Who Are Overweight (%)^b^	Combined No. Who Are at Risk for Overweight and Overweight (%)^c^
**Combined**			
Third (n = 71)	30 (42.2)	32 (45.1)	41 (57.8)
Fourth (n = 60)	24 (40.0)	30 (50.0)	36 (60.0)
Fifth (n = 67)	27 (40.3)	33 (49.3)	40 (59.7)
Seventh (n = 29)	10 (34.5)	11 (37.9)	19 (65.5)
Eighth (n = 24)	11 (45.8)	10 (41.7)	13 (54.2)
Total (n = 251)	102 (40.6)	116 (46.2)	149 (59.4)
**Boys**			
Third (n = 36)	16 (44.4)	15 (41.7)	20 (55.6)
Fourth (n = 30)	10 (33.3)	16 (53.3)	20 (66.7)
Fifth (n = 32)	11 (34.4)	19 (59.4)	21 (65.6)
Seventh (n = 17)	5 (29.4)	9 (52.9)	12 (70.6)
Eighth (n = 14)	7 (50.0)	6 (42.9)	7 (50.0)
Total (n = 129)	49 (38.0)	65 (50.4)	80 (62.0)
**Girls**			
Third (n = 35)	14 (40.0)	17 (48.6)	21 (60.0)
Fourth (n = 30)	14 (46.7)	14 (46.7)	16 (53.3)
Fifth (n = 35)	16 (45.7)	14 (40.0)	19 (54.3)
Seventh (n = 12)	5 (41.7)	2 (16.7)	7 (58.3)
Eighth (n = 10)	4 (40.0)	4 (40.0)	6 (60.0)
Total (n = 122)	53 (43.4)	51 (41.8)	69 (56.6)

Abbreviation: BMI, body mass index.

a Prevalence for age- and sex-adjusted BMI <85th percentile (normal weight), which may include children who were underweight.

b Prevalence for age- and sex-adjusted BMI ≥95th percentile (overweight).

c Prevalence for age- and sex-adjusted BMI ≥85th percentile (at risk for overweight combined with overweight).
